# Increased extracellular volume in the liver of pediatric Fontan patients

**DOI:** 10.1186/s12968-019-0545-4

**Published:** 2019-07-15

**Authors:** Charlotte de Lange, Marjolein J. E. Reichert, Joseph J. Pagano, Mike Seed, Shi-Joon Yoo, Craig S. Broberg, Christopher Z. Lam, Lars Grosse-Wortmann

**Affiliations:** 1 Department of Pediatrics, Division of Cardiology, The Hospital for Sick Children, University of Toronto, Ontario, Canada; 2 Division of Radiology and Nuclear medicine, Pediatric section, Rikshospitalet, Oslo University Hospital, Oslo, Norway; 3 Department of Pediatrics, Division of Cardiology, Stollery Children’s Hospital, University of Alberta, Edmonton, Alberta Canada; 4 Department of Diagnostic Imaging, The Hospital for Sick Children, University of Toronto, Toronto, Ontario Canada; 5 Knight Cardiovascular Institute, Division of Cardiovascular Medicine, Oregon Health and Science University, Portland, Oregon, USA

**Keywords:** Cardiovascular magnetic resonance, Fontan circulation, Liver cirrhosis, T1 mapping, Single ventricle

## Abstract

**Background:**

Patients with single ventricle physiology are at increased risk for developing liver fibrosis. Its extent and prevalence in children with bidirectional cavopulmonary connection (BCPC) and Fontan circulation are unclear. Extracellular volume fraction (ECV), derived from cardiovascular magnetic resonance (CMR) and T1 relaxometry, reflect fibrotic remodeling and/or congestion in the liver. The aim of this study was to investigate whether pediatric patients with single ventricle physiology experience increased native T1 and ECV as markers of liver fibrosis/congestion.

**Methods:**

Hepatic native T1 times and ECV, using a cardiac short axis modified Look-Locker inversion recovery sequence displaying the liver, were measured retrospectively in children with BCPC- and Fontan circulations and compared to pediatric controls.

**Results:**

Hepatic native T1 time were increased in Fontan patients (*n* = 62, 11.4 ± 4.4 years, T1 762 ± 64 ms) versus BCPC patients (*n* = 20, 2.8 ± 0.9 years, T1 645 ± 43 ms, *p* = 0.04). Both cohorts had higher T1 than controls (*n* = 44, 13.7 ± 2.9 years, T1 604 ± 54 ms, *p* < 0.001 for both). ECV was 41.4 ± 4.8% in Fontan and 36.4 ± 4.8% in BCPC patients, respectively (*p* = 0.02). In Fontan patients, T1 values correlated with exposure to cardiopulmonary bypass time (R = 0.3, p = 0.02), systolic and end diastolic volumes (R = 0.3, p = 0.04 for both) and inversely with oxygen saturations and body surface area (R = -0.3, p = 0.04 for both). There were no demonstrable associations of T1 or ECV with central venous pressure or age after Fontan.

**Conclusion:**

Fontan and BCPC patients have elevated CMR markers suggestive of hepatic fibrosis and/or congestion, even at a young age. The tissue changes do not appear to be related to central venous pressures.

**Trial registration:**

Retrospectively registered data.

**Electronic supplementary material:**

The online version of this article (10.1186/s12968-019-0545-4) contains supplementary material, which is available to authorized users.

## Introduction

The Fontan operation, introduced over 40 years ago, and its permutations place the systemic and pulmonary circulations in series rather than in parallel in patients with functionally univentricular hearts [1, 2]. Fontan and bidirectional cavopulmonary connection (BCPC) hemodynamics depend on a central venous pressure that is high enough to overcome the pulmonary vascular resistance. The ensuing systemic venous congestion and a limited ability to augment cardiac output result in end-organ dysfunction, affecting the liver, intestine, and other organ systems [3] . Fontan associated liver disease (FALD) with hepatic congestion and fibrotic remodeling are increasingly recognized [4]. However, the extent of hepatic fibrosis as well as its potential causes and clinical significance are incompletely understood, partially due to the current difficulties in monitoring tissue fibrosis non-invasively.

Native T1 times and extracellular volume fraction (ECV) derived from cardiovascular magnetic resonance (CMR) T1 relaxometry, often referred to as ‘T1 mapping’, are non-invasive markers of diffuse tissue fibrosis increasingly applied to the myocardium [5–9]. More recently, CMR T1 mapping of the liver has been employed to quantify the severity of chronic liver disease in adults without congenital heart disease, yielding a good correlation to the histological severity of fibrotic remodeling [10–12].

In the present study we aimed to investigate the feasibility of T1 mapping in the liver in pediatric Fontan and BCPC patients on routinely performed CMR. We hypothesized that CMR markers of extracellular volume (ECV) expansion are increased in patients with single ventricle physiology and associated with their hemodynamics.

## Methods

In this retrospective cross-sectional cohort study, consecutive pediatric patients with BCPC and Fontan-type palliations who underwent CMR between April 2014 and March 2018 were included. During that interval T1 relaxometry was routinely included in the CMR protocol. Patients with a non-diagnostic T1 relaxometry acquisition pertaining to the liver were excluded. Only the latest exam was included when more than one CMR scan were eligible.

Demographic, clinical, and surgical Information were retrieved from patient charts. Cumulative cardiopulmonary bypass- (CPBT), circulatory arrest- (CAT) and cross-clamp times (CCT) were recorded. The patients’ oxygen saturations and blood pressures at the time of CMR (prior to general anesthesia, if applied), serum biomarkers of hepatic function and hemodynamic data from cardiac catheterization as long as performed within 6 months of the CMR, were also retrieved.

Synthetic liver dysfunction was defined as an albumin concentration below the normal range in patients without protein losing enteropathy (PLE) and/or an elevation of the international normalized ratio (INR) above 1.2 in patients who were not receiving warfarin. Metabolic liver dysfunction was indicated by any of the following values outside of the normal range for age: unconjugated bilirubin, aspartate transaminase, alanine aminotransferase, alkaline phosphatase, gamma-glutamyl transferase, lactate dehydrogenase.

As a control group, patients examined between April 2014 and March 2017, with the same T1 relaxometry protocol for a family history of arrhythmogenic right ventricular cardiomyopathy or non-specific chest pain referred for anatomical coronary artery imaging were included, as long as their entire work-up was negative.

### Cardiovascular magnetic resonance imaging

All patients and controls underwent CMR examinations on a 1.5 T system (‘Magnetom Avanto’, Siemens Healthineers, Erlangen, Germany). The detailed protocol has been published elsewhere [9]. In brief, a modified Look-Locker inversion recovery (MOLLI) sequence at a single cardiac short axis slice was performed before and 15 min after injection of a gadolinium-based contrast agent, 0.2 mmol/kg of either Gadobuturol (‘Gadavist’, Bayer Healthcare, Berlin, Germany) or gadobenate dimeglumine (‘Multihance’, Bracco S.p.A., Milan, Italy). A stack of balanced steady-state-free-precession short axis cine loops were obtained for volumetric measurements, in addition to through plane phase contrast cine acquisitions for blood flow measurements of the ascending and descending aorta and the pulmonary arteries and veins.

### Image post-processing

All T1 mapping analyses were conducted by a single observer (CdL). T1 relaxation times were extracted from the MOLLI acquisitions using commercially available software (‘Qmap’ in ‘MedisSuite‘version 2.1, MEDIS Medical Imaging Systems, Leiden, The Netherlands): Corresponding regions of interest (ROIs) were drawn on each of the eight motion corrected source images. T1 times were derived based on post-inversion recovery times and signal intensities, using a curve fitting algorithm.

**Fig. 1:**
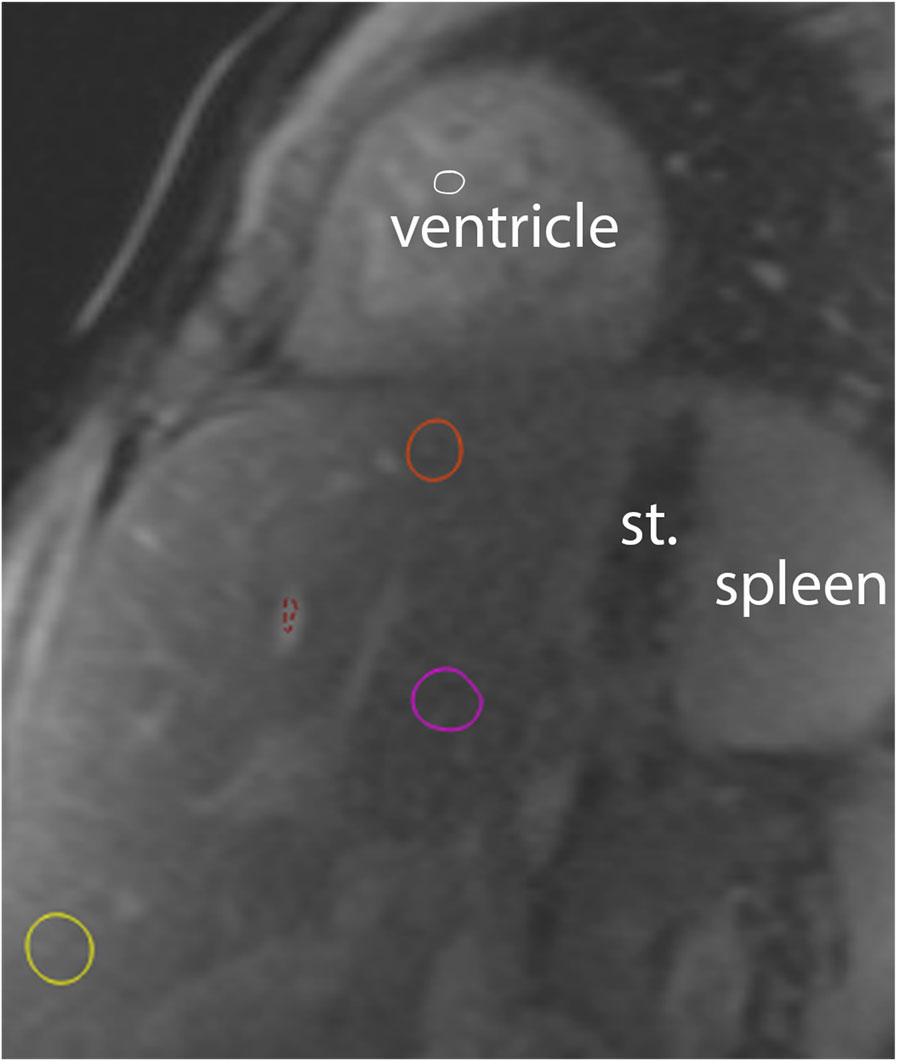
Hepatic Native T1. Native T1 source image in short-axis orientation demonstrating the heart and upper part of the abdomen. Regions of interest (ROIs) depict liver regions near diaphragm (#1, orange), central (#2, pink) and caudal (#3, yellow). The red ROI samples signal in the blood pool of a liver vein and the white ROI (#5) in the ventricular cavity. Stomach (st)

Portions of the right and left lobes of the liver were visible on the mid-ventricular short axis slice. T1 relaxation times were measured by tracing three regions of interest (ROI): ROI 1, near the diaphragm, ROI 2, in the middle near the hepatic hilum, and ROI 3, in the caudal and peripheral part of the liver. All ROIs were traced to avoid major vessels, the gall bladder and hepatic surface (Fig. 1) [13, 14]. For blood pool T1 measurements, ROIs were placed in the cardiac lumen and in a hepatic vein as close as possible to the diaphragm, while avoiding myocardial trabeculations and hepatic tissue, respectively. All T1 measurements were corrected for incomplete inversion during the MOLLI acquisitions [15]. The ECV of all liver ROIs were calculated based on the pre- and post-contrast T1 values. For the blood pool T1 values, a 75/ 25% split between hepatic venous blood and cardiac lumen blood was used, given that 75% of hepatic blood flow is derived from the portal vein, i.e. is deoxygenated, similar to the hepatic venous blood. Hematocrit values were sampled within one week of the CMR examination (typically on the same day), and ECV was calculated according to the following formula :

$$ ECV=\left(1- haematocrit\right)\frac{\left(1/ postcontrast\mathrm{T}1 liver\right)-\left(1/ native\mathrm{T}1 liver\right)}{\left(1/ postcontrast\mathrm{T}1 blood\right)-\left(1/ native\mathrm{T}1 blood\right)} $$

A randomly selected sample of 20 patients was contoured by a second observer (MR) for observer variability. The same observer re-analyzed the same sample after an interval of at least 5 weeks blinded to the original results.

The same software platform (‘MedisSuite’, ‘Qmass’ module) was used for the myocardial volumetric measurements: both ventricular chambers were contoured as one chamber as long as they were connected via a ventricular septal defect (VSD) and / or both chambers ejected into the (neo-) aorta. Otherwise, only the dominant ventricle was contoured.

In addition, flow in the ascending and descending aorta, superior vena cava and the pulmonary arteries and veins were analyzed. Aorto-pulmonary collateral (APC) flow was calculated by subtracting total pulmonary arterial flow from the sum of the pulmonary venous flows.

### Statistical analysis

Continuous variables are presented as means ± standard deviation (SD) if normally distributed and otherwise as medians and ranges. Categorical variables are expressed as counts and percentages of the total. Student’s t tests, Wilcoxon Rank-Sum tests or and analysis of variance (ANOVA) with post-hoc Tukey test for multiple comparisons, when appropriate, were used for comparisons between patient groups for continuous variables and Fisher’s exact tests for categorical variables.

Correlations between continuous and categorical parameters were assessed using univariate regression analysis. A multivariate regression model was performed for variables with a significant number of patients and with *p*-values < 0.01 on univariate testing. *P*-values < 0.05 were considered statistically significant. Intra- and inter observer reproducibility’s were assessed by Bland-Altman analyses [16]. Statistical analyses were performed using SPSS version 25 (Statistical Package for the Social Sciences (SSPS) International Business Machines, Inc., Armonk, New York, USA).

## Results

### Patient characteristics

Patient characteristics including clinical parameters are presented in Table 1. Of 83 identified patients that met the inclusion criteria, 20 with BCPC (2.8 ± 0.9 years, range 0.6–4.5) and 62 with Fontan (11.4 ± 4.4 years, range 3.6–17.7) were included. One patient was excluded because of a non-diagnostic MOLLI sequence in whom only a small area of the liver was shown on the MOLLI images. In the Fontan group, there were 34 patients with a dominant single left ventricle (SLV) and 21 with a dominant single right ventricle (SRV). Seven patients had two good-sized ventricles and were excluded from analyses comparing SRV and SLV. In the BCPC group there were six patients with a SLV and ten with a SRV. Two patients had two good-sized ventricles and two had one single ventricle with unidentified morphological features. Forty-four individuals (age 13.9 ± 2.7 years, range 9–18) constituted the control group.

**Table 1:** Patient demographics, surgical and catheterization results, presented as mean values and standard deviations, with ranges in brackets as appropriate

	Control *n* = 44	BCPC *n* = 20	Fontan *n* = 62	*p* value BCPC vs control	*p* value Fontan vs control	*p* value Fontan vs BCPC	SLV Fontan *n* = 34	SRV Fontan *n* = 21	*p* value SRV vs SLV
**Clinical**									
age at CMR (years)	13.7 ± 2.9 (7.4–17.7)	2.8 ± 0.9 (0.6–4.5)	11.4 ± 4.4 (3.6–17.8)	< 0.001*	0.001*	< 0.001*	12.2 ± 4.2	11.4 ± 4.2	0.3
gender, male n (%)	18 (41%)	13 (65%)	36 (58%)	0.1	0.06	0.4	21 (61%)	11 (52%)	0.8
weight (kg)	59.9 ± 23.3	13.3 ± 2.8	37.1 ± 17.6	< 0.001*	< 0.001*	< 0.001*	41.5 ± 18.5	36.3 ± 15.1	0.2
BSA (m^2^)	1.6 ± 0.38	0.6 ± 0.1	1.2 ± 0.38	< 0.001*	< 0.001*	< 0.001*	1.3 ± 0.4	1.2 ± 0.4	0.5
systolic BP (percentile)	–	85.8 ± 23.1	60.7 ± 29.3	–	–	**< 0.001**	58.8 ± 30.2	60.6 ± 28.5	0.8
diastolic BP (percentile)	–	81.7 ± 17.8	49.2 ± 28.4	–	–	**< 0.001**	50.3 ± 29.3	41.9 ± 23.9	0.2
O_2_ saturation (%)	–	84 ± 5	93 ± 5	–	–	**< 0.001**	94 ± 5	93 ± 5	0.6
**Surgical**									
age at BCPC (years)	–	0.5 ± 0.2	0.6 ± 0.4	–	**–**	0.3	–	–	
age at Fontan (years)	–	–	3.3 ± 0.9	–	**–**	**–**	3.2 ± 0.8	3.5 ± 1.1	0.2
interval MR BCPC/ Fontan op (days)	–	3895 ± 658	2888 ± 1684	–	**–**	**0.01**	3296 ± 1619	2856 ± 1628	0.3
total CPBT (minutes)	–	154 ± 98	252 ± 108	–	–	**0.001**	209 ± 107	310 ± 75	**0.001**
total CAT (minutes)	–	26 ± 9	16 ± 15	–	–	0.08	20.5 ± 23	16 ± 14	0.7
total CCT (minutes)	–	62 ± 56	82 ± 64	–	–	0.2	60 ± 66	97 ± 42	**0.03**
**Catheterization**									
interval MR to catheterization (days)	–	86 ± 183	1719 ± 183	–	–	**< 0.001**	2096 ± 1898	1675 ± 726	0.4
age at catheterization (years)	–	2.6 ± 1.2	6.7 ± 4.2	–	–		6.8 ± 4.7	6.9 ± 3.9	0.9
VEDP (mmHg)	–	7.7 ± 2.1	7.2 ± 2.3	–	–	0.6	8.3 ± 2.3	6.3 ± 2.6	0.2
CVP (mmHg)	–	10.7 ± 3.1	10.9 ± 0.8	–	–	0.6	10.3 ± 1.5	10.6 ± 1.7	0.6
atrial pressure (mmHg)	–	4.9 ± 2.1	5.3 ± 2.1	–	–	0.6	6.2 ± 2.3	5.4 ± 1.1	0.5

*One way Analysis of variance tested for multiple comparisons with post-hoc Tukey

*BCPC bidirectional cavo-pulmonary connection, BSA body surface area, BP blood pressure, CAT circulatory arrest time, CPBT cardiopulmonary bypass time; CCT cross-clamp time, CMR cardiovascular magnetic resonance, CVP central venous pressure, SLV single left ventricle; SRV single right ventricle, VEDP ventricular end-diastolic pressure*

Serum liver function testing was available in 31 Fontan patients and only two had all hepatic biomarkers within the normal range. None of the patients had severe metabolic or synthetic dysfunction. Twenty-three of 31 Fontan patients (74%) had mildly abnormal parameters of metabolic hepatic function as evidenced by bilirubin, alkaline phosphatase, aspartate aminotransferase, lactate dehydrogenase and gamma-glutamyl transferase. Fourteen of 27 Fontan patients (52%) had mildly deranged synthetic function with increase in INR (up to 1.8) and decrease in albumin (24–46 g/L, normal values 35–52 g/L).

### T1 and extracellular volume fraction

Native T1 and ECV differed between the three ROIs (Additional file 1: Table S1). Given these differences, the average T1 and ECV from ROIs 1–3 were chosen as the most representative values for the liver and used for all subsequent analyses.

Blood pool native T1 values, were significantly higher in the ventricular blood pool than in hepatic venous blood, for controls (1559 ± 74 and 1367 ± 252 ms respectively, *p* < 0.001) and for patients (1590 ± 112 versus 1427 ± 207 ms respectively, (*p* < 0.001). This difference was attributed to the lower oxygen saturations in hepatic venous as compared to systemic ventricular blood.

Thirty-two out of 82 patients received gadobenate dimeglumine (‘Multihance’) and the remaining 50 patients (33 Fontan and 17 BCPC patients) had gadobuturol (‘Gadavist’). Of the 19 controls who received gadolinium, 15 had gadobenate dimeglumine and four gadobuturol. Liver ECV was markedly higher in subjects who had received gadobenate diglumine as compared to gadobuturol: 45.4 ± 4.0% vs. 39.8 ± 5.2% (*p* < 0.001) for patients (BCPC and Fontan combined) and 58.1 ± 12.6% and 32.1 ± 4.8% (*p* < 0.001) for controls. For controls, the two groups did not differ significantly with regards to their gender, age composition or their hepatic native T1 times (620 ± 48 and 610 ± 71 ms, respectively, *p* = 0.8). We suspect that the ECV measurements in the gadobenate dimeglumine group were falsely elevated due to the partial biliary excretion (3–5%) of the drug, and consequently, ECV was computed only for patients who had received gadobuturol.

**Fig. 2:**
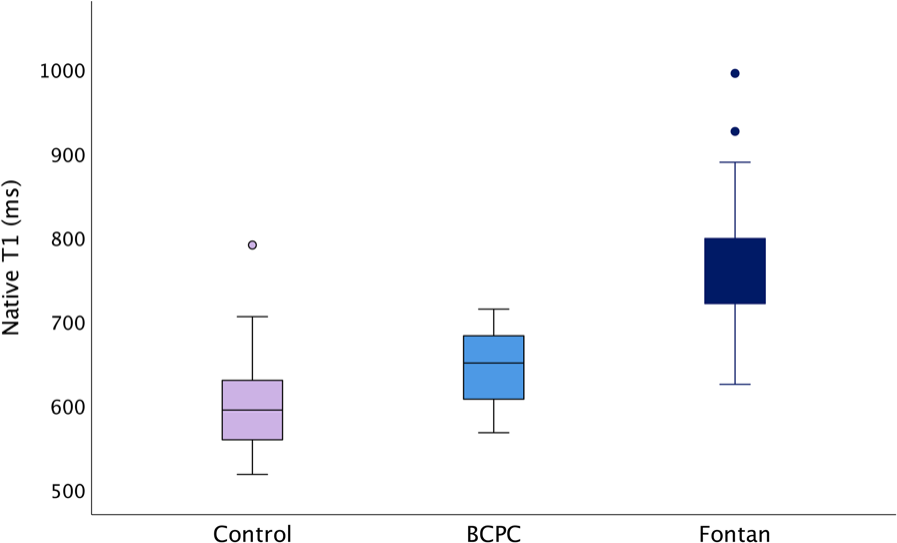
Boxplot of native T1 values. Boxplot of native T1 values in the Fontan, bidirectional cavopulmonary connection (BCPC) and control groups

Native T1 times in BCPC and Fontan patients were elevated as compared to controls, (*p* < 0.001 for both groups) (Fig. 2 and Table 2). Native T1 and ECV were higher in Fontan than in BCPC patients (*p* = 0.04 for native T1 and ECV *p* = 0.02, respectively). ECV was not significantly different for SLV and SRV (*p* = 0.1) (Table 2). BCPC patients with SRVs had lower T1 values than SRV Fontan patients (666 ± 41  versus 780 ± 78 ms, *p* = < 0.001). Both T1 and ECV were lower in SLV BCPC patients than in SLV Fontan patients (642 ± 37 versus 748 ± 51 ms, *p* < 0.001 and (36.1 ± 4.8% versus 41.7 ± 5.1%, *p* = 0.001, respectively).

**Table 2:** T1 relaxometry, ventricular volumetry, blood flow volumes

	Control *n* = 44	BCPC *n* = 20	Fontan *n* = 62	*p* value BCPC vs control	*p* value Fontan vs control	*p* value Fontan vs BCPC	SLV Fontan. *n* = 34	SRV Fontan *n* = 21	*p* value SRV vs SLV
VEDVi (mL/m^2^)	93 ± 13	135 ± 30	127 ± 41	< 0.001*	< 0.001*	0.4*	119 ± 35	144 ± 49	**0.03**
VESVi (mL/m^2^)	39 ± 7	76 ± 22	72 ± 31	< 0.001*	< 0.001*	0.5*	64 ± 22	88 ± 40	**0.05**
VSVi (mL/m^2^)	54 ± 8.9	59.1 ± 14.3	55.4 ± 16.3	0.2*	0.6*	0.3*	55.7 ± 19.0	56.3 ± 12.3	0.9
Vmass (g/m^2^)	55 ± 12	59 ± 15	57 ± 9	0.3*	0.5*	0.4*	59 ± 10	53 ± 10	**0.02**
VEF (%)	58 ± 5	44 ± 7	45 ± 8	< 0.001*	< 0.001*	0.7*	47 ± 8	40 ± 8	**0.009**
Vmass / VEDV (g/mL)	–	78 ± 19	71 ± 21	–	–	0.1	70 ± 20	76 ± 25	0.3
AAO (L/min/m^2^)	–	4.2 ± 1.4	3.3 ± 0.7	–	–	**0.01**	3.2 ± 0.6	3.4 ± 0.9	0.4
APC (L/min/m^2^)	–	1.2 ± 0.7	0.7 ± 0.5	–	–	**0.004**	0.6 ± 0.4	0.7 ± 0.5	0.7
Qp/Qs	–	1.2 ± 0.4	1.1 ± 0.2	–	–	0.5	1.1 ± 0.2	1.1 ± 0.1	0.5
Hepatic native T1 (ms)	604 ± 54	645 ± 43	762 ± 64	< 0.001*	< 0.001*	0.04*	748 ± 51	780 ± 78	0.07
Hepatic ECV (%) *n* = 50 (with Gadobuturol)	–	36.4 ± 4.8 *n* = 17	41.4 ± 4.8 *n* = 33			**0.02**	40.2 ± 4.2	43.1 ± 4.7	0.1

*One way Analysis of variance tested for multiple comparisons with post-hoc Tukey

*AAO ascending aortic flow, APC aorto-pulmonary collateral flow, BCPC bidirectional cavopulmonary connection, DAO descending aorta, ECV extracellular volume fraction, Qp pulmonary (venous) blood flow, Qs systemic blood flow, SLV single left ventricle, SRV single right ventricle SVC superior vena cava, VEDVi indexed ventricular end diastolic volume, VEF ventricular ejection fraction, VESVi indexed ventricular end systolic volume, Vmassi indexed ventricular mass, VSVi indexed ventricular stroke volume*

The comparisons between Fontan patients with normal and abnormal hepatic (synthetic or metabolic) hepatic function regarding T1 and ECV were underpowered due to the low number of patients with available biochemistry data.

### Association between T1, ECV and clinical parameters

In Fontan patients, T1 values correlated weakly with the sum of all previous CPBTs (R = 0.3 *p* = 0.02, CI (0.04–0,40)), with total CCT (R = 0.3 *p* = 0.03, CI (0.00–0.56)) and with total CAT (R = 0.3 p = 0.02, CI (-1.17 − 3.85)). Native T1 correlated with end-systolic and end-diastolic volumes (R = 0.3, *p* = 0.04 for both, CI (0.01–1.06) and (0.01–0.8) respectively) and with APC flow (R = 0.3, *p* = 0.01, CI (9.2–77.5)). Native T1 correlated inversely with SaO_2_ and body surface area (R = -0.3, p = 0.04 for both, CI (-6.63 − -0.26) and (-89.50 − -3.35) respectively).

There were no correlations of liver T1 or ECV with ventricular end-diastolic, and central venous pressures. However, the statistical power for these analyses was low due to the modest sample size. Patients with a patent Fontan fenestration (*n* = 44, 70%) had similar T1 and ECV as the remainder of Fontan patients.

### Reproducibility

**Fig. 3:**
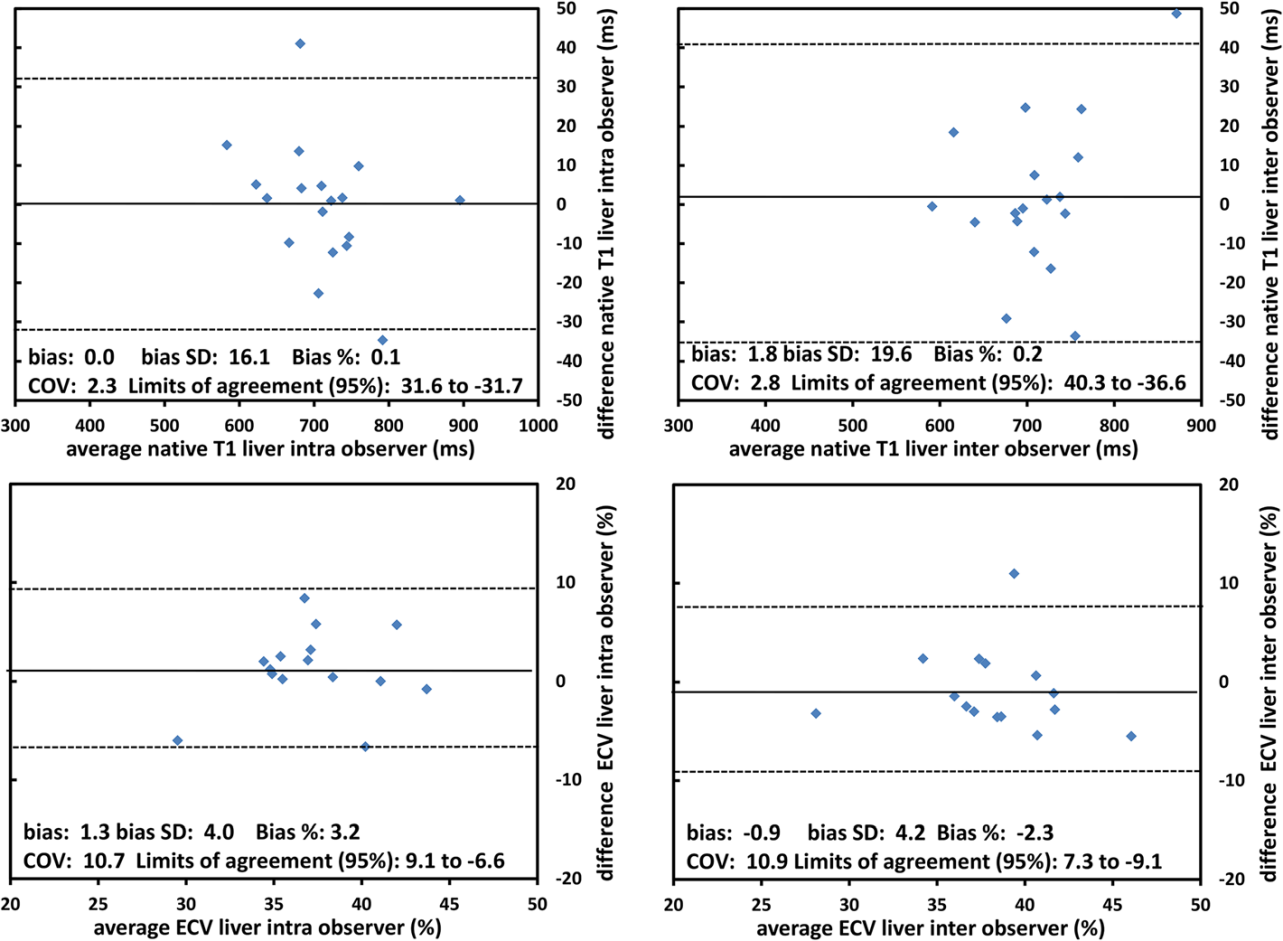
Reproducibility of T1 and ECV. Bland Altman plots for intra and inter observer agreement for measurements of native T1 (upper row) and Extracellular volume fraction (ECV, lower row) for the average of ROIs 1–3

Reproducibility was tested with Bland Altman plots for the T1 and ECV values of ROI 1–3. Native T1 and ECV demonstrated moderate intra- and inter-observer variation, with a relative bias of 0.1 and 0.2%, respectively for T1 and 3.2% and -2.3%, respectively for ECV (Fig. 3). Coefficients of variation were 2.3 and 2.8, respectively, for T1 and 10.7 and 10.9, respectively, for ECV. The reproducibility metrics of the individual ROIs are presented in Additional file 1: Table S1. In summary, the average of ROI 1–3 values which were considered the most representative for the displayed section of the liver tissue, revealed the best reproducibility.

## Discussion

Liver fibrosis is an increasingly recognized complication in older Fontan patients but little is known about the severity of hepatic fibrotic remodeling in young individuals with single ventricle physiology. CMR T1 relaxometry presents an opportunity to measure the degree of extracellular volume expansion non-invasively. The current study helped us refine the technique of hepatic T1 relaxometry, shaping the methods in future studies. Our results offer the following novel insights into hepatic tissue changes:

1. CMR T1 mapping is feasible in the liver of young patients with congenital heart disease.
2. Imaging biomarkers of hepatic diffuse fibrosis and/or congestion are elevated in young patients with single ventricle physiology, especially in those with a Fontan palliation.
3. Native T1 and ECV values are associated with exposure to cardiopulmonary bypass.
4. Technical advances from this study regarding hepatic ECV quantification are that a) gadolinium contrast agent with even partial biliary excretion must be avoided, b) hepatic venous and left ventricular blood pool T1values differ and c) averaging multiple ROIs is a suitable strategy to characterize liver ECV and T1 in light of heterogeneously distributed tissue changes in the Fontan population.

### Fontan associated liver disease (FALD)

FALD constitutes important morbidity in patients with functionally single ventricles. It affects candidacy for cardiac transplantation and other palliative operations. Furthermore, liver cirrhosis predisposes to malignant transformation of liver tissue to hepatocellular carcinoma [3, 17–21]. Early fibrotic remodeling, on the other hand, may be reversible and present a window for therapeutic intervention.

The challenge in monitoring hepatic fibrosis until recently lay within the lack of non-invasive techniques to detect and quantify the degree of remodeling. Liver biopsy is the diagnostic gold standard but invasive and not without risk. As such, it does not lend itself to serial examinations. Furthermore, due to the heterogeneous hepatic changes the sampled tissue may not be representative of the average degree of fibrosis [22, 23]. More recently, ‘stiffness’ by elastography, based on either ultrasonography (USE) or magnetic resonance elastography (MRE), has assumed a role in the non-invasive evaluation of chronic liver disease, including in Fontan patients [21, 24–27]. However, the limitations of elastography are that it a) is an indirect measure of tissue behavior rather than a direct marker of extracellular volume expansion, b) does not distinguish between fibrosis and congestion, c) has not been demonstrated to predict outcome, and d) requires additional hardware not widely available.

### T1 and ECV are elevated in BCPC and Fontan patients

CMR T1 relaxometry overcomes some of these limitations by providing non-invasive, quantitative tissue characterization with a spatial resolution in the millimeter range. In non-congestive adult chronic liver disease, native T1 times can discriminate between histologic fibrotic grades [10, 28, 29] and may predict outcomes [11, 30].

Only pilot data in 16 adult Fontan patients yielded CMR T1 and ECV levels comparable to our cohort [14]. In our study, Fontan patients demonstrated elevated native T1 times, in line with results from USE, computed tomography and postmortem studies which have indicated increased stiffness and evidence of fibrosis [31–34]. Fontan patients had higher T1 and ECV than BCPC patients, suggesting an increase in fibrosis/ congestion as patients transition to the Fontan circulation. Whether this is true in individual patients remains to be confirmed with serial CMRs. A recent USE study revealed a transient increase in hepatic stiffness soon after the Fontan operation, leading the authors to hypothesize that in this scenario stiffness represents congestion, rather than fibrosis which would take longer to develop [26]. Our results did not reveal an association of either T1 or ECV with central venous pressure (CVP). However, these analyses were poorly powered due to the modest sample size. Like elastography, T1 relaxometry does not distinguish unequivocally between fibrosis, congestion or inflammation, as all elevate both T1 and ECV; it seems plausible that all three mechanisms contribute to the abnormal T1 and ECV values. In the future, adding T2 mapping and T2 weighted imaging may prove helpful in distinguishing between fibrosis and edema. Further, T1rho relaxation time may become useful in monitoring liver fibrosis and in distinguishing it from fat, edema and/or inflammation [22, 35, 36]. Despite an uncertainty about the exact histological correlate, monitoring markers of extracellular matrix expansion may prove beneficial, given that congestion is regarded as a precursor of FALD [37–39]. Nonetheless, the prognostic significance of liver T1 relaxometry remains to be determined.

Our results suggest that the etiology of hepatic changes is multifactorial: For example, exposure to cardiopulmonary bypass is associated not only with long-term myocardial, but also hepatic remodeling [9, 40]. Another candidate risk factor for hepatic remodeling are decreased oxygen delivery as a result of hypoxemia and low cardiac output in this population. An association between CVP and liver stiffness was mostly described in older Fontan patient populations. In our young cohort, the other potential factors may be relatively more important than CVP. In this context, it is interesting to note that T1 and ECV did not increase with age, also pointing to factors other than chronic venous congestion as the principal pathomechanism [34].

Fontan patients with an SRV revealed a trend towards higher liver T1 values than those with a SLV. Whether this difference is related to longer bypass and cross-clamp times, the worse ventricular performance or a less obvious risk factor in the SRV group remains to be determined.

### T1, ECV and liver function

In keeping with other pediatric reports, markers of hepatic function revealed only mild perturbations of synthetic and metabolic performance in 52 and 74%, respectively, of the Fontan patients who underwent biochemistry testing. No reliable analysis of an association of serum markers with ECV or T1 could be performed due to small sample size. Previous histological studies highlight the poor reflection of early liver injury in serum markers, due to the liver’s remarkable ability to compensate for these tissue changes until their late stages [19, 33].

### Liver T1 Relaxometry - technical aspects

The current study yields several important technical insights into liver T1 mapping and ECV analysis. Most importantly, gadolinium agents with even a small percentage of biliary secretion, such as gadobenate dimeglumine, are not suitable for the quantification of ECV because their concentration in the bile system falsely decreases post-contrast T1, leading to erroneously high ECV results.

In contrast to previously published reports [14] our data suggest that the blood pool T1 producing the most representative hepatic ECV measurements is not the ventricular cavity which is used for myocardial analyses. We found systematically found lower pre-contrast T1 times in the liver vein as compared to the heart. The principal cause of this discrepancy is likely the lower oxygen saturations in hepatic venous as compared to ventricular blood, as T1 is lower in less oxygenated blood [41]. In the liver, blood flow is mainly provided by the portal vein in about 75% while 25% is provided by the hepatic artery [39]. Therefore, performing the ECV calculations using a 75/25% split between hepatic venous blood and arterial cardiac lumen blood can be expected to produce a more ‘true’ hepatic ECV value.

Liver fibrosis is known to be a geographically heterogeneous process. As a result, we found systematic regional differences in the sampled T1. Previous studies implied that portal venous hypertension progresses centripetally from the liver periphery [38, 39]. As such, one would expect lower fibrosis/congestion markers near the center (ROI1) which was true in our cohort. Given the observed regional variation of tissue composition along with the superior reproducibility of averaging multiple ROIs, sampling T1 in multiple regions is the preferred approach in the Fontan population [13, 14, 29]. Sampling a large ROI encompassing the entire liver in the field of view is problematic as it is challenging to avoid medium sized hepatic vessels.

The technical challenges with hepatic MR relaxometry pertain mostly to ECV and its calculation. In the present study, ECV did not appear to add insights beyond what was apparent from native T1 analysis. Relying solely on T1 and foregoing ECV is attractive as it avoids the need for intravenous gadolinium as well as abbreviate scan and post-processing times. On the other hand, ECV has been demonstrated to be more comparable across scanner manufacturers, field strengths, and patient populations [37, 42]. It is too early to conclude whether liver MR relaxometry should be performed using native T1, ECV or both.

### Study limitations

Several limitations of this study warrant discussion: 1.) Since, most control subjects received gadobenate dimeglumine which is biliarily excreted, there was no control group for hepatic ECV. 2.) The pediatric control group was older than the patient group, limiting the comparability between the groups. Furthermore, the BCPC group was more homogeneous in age than the Fontan group which may have accounted for broader range of T1 and ECV values among Fontan patients. This relatively wide spectrum may have obscured some of the differences between Fontan patients and controls. 3.) It is possible that postprandial conditions, not controlled for during this study, influences native T1 and ECV owing to differences in portal venous flow [43]. 4.) Given the heterogeneity of hepatic remodeling the section of the liver that was included in the short axis MOLLI slice may not be representative of the entire liver parenchyma. For prospective studies, dedicated T1 acquisitions of the liver are advised. While it would be preferable to obtain the T1 value for the entire portion of the liver displayed in the image this was not feasible due to the signal from blood vessels in the image which would have contaminated the T1 values and could not be excluded by thresholding due to imperfect co-registration of the source images despite motion correction. 5.) It is unclear whether axial liver T1 mapping acquisitions would have yielded different results from the short axis sequences that were available for review. 6.) Even though we used a blood pool signal with an oxygen saturation that approximated that within the liver parenchyma for T1, the resultant ECV may differ from the true liver values. 7.) Low albumin, which was used as a marker of hepatic synthetic insufficiency, also occurs in PLE. While this parameter was not evaluated in patients with overt PLE, an intestinal protein leak can be subclinical in Fontan patients. 8.) The retrospective study design may have introduced a selection bias and caused gaps in some of the results, most notably in the liver serum biomarkers, which were available only in a subgroup of patients. 9.) Finally, while the cross-sectional information presented here suggests that fibrosis / congestion markers increase with the transition from the BCPC to the Fontan circulation only longitudinal data can elucidate the development of these changes in individual patients over time*.*

## Conclusion

In this study of a large cohort of pediatric patients with single ventricle circulations, imaging biomarkers of fibrosis and/or congestion are elevated and particularly after the Fontan operation. These liver tissue changes are not clearly associated with Fontan pressure or ventricular systolic performance. Longitudinal studies are required to test the prognostic significance of these derangements. Pitfalls of ECV quantification are related to the use of gadolinium contrast with biliary excretion, blood pool T1 sampling regions, and the heterogeneity of liver remodeling.

## Additional file

Additional file 1: **Table S1.** Inter region T1, ECV differences. Native T1 times and extracellular volume fraction (ECV) for the different ROIs 1–3 and the average of ROIs 1–3 with observer variation. (DOC 41 kb)

## Data Availability

The datasets used and/or analyzed during the current study are available from the corresponding author on reasonable request.

## Abbreviations

ANOVA: Analysis of variance
APC: Aortopulmonary collateral
BCPC: Bidirectional cavopulmonary connection
CAT: Circulatory arrest time
CCT: Cross-clamp time
CMR: Cardiovascular magnetic resonance
CPBT: Cardiopulmonary bypass time
CVP: Central venous pressure
ECV: Extracellular volume fraction
EF: Ejection fraction
FALD: Fontan associated liver disease
INR: International normalized ratio
LGE: Late gadolinium enhancement
MOLLI: Modified Look-Locker inversion recovery
MRE: Magnetic resonance elastography
PLE: Protein losing enteropathy
ROI: Region of interest
SD: Standard deviation
SLV: Single left ventricle
SRV: Single right ventricle
SV: Single ventricle
USE: Ultrasonography elastography
VEDP: Ventricular end diastolic pressure
VEDV: Ventricular end diastolic volume
VESV: Ventricular end systolic volume
VSD: Ventricular septal defect
