# Supplementary material for: Increased extracellular volume in the liver of pediatric Fontan patients
Source: J Cardiovasc Magn Reson. 2019 Jul 15;21:39. doi: 10.1186/s12968-019-0545-4 (PMC6628496; doi:10.1186/s12968-019-0545-4)
Supplement: Supplementary file 1 — Table S1. Inter region T1, ECV differences. Native T1 times and extracellular volume fraction (ECV) for the different ROIs 1–3 and the average of ROIs 1–3 with observer variation. (DOC 41 kb) [file 12968_2019_545_MOESM1_ESM.doc]

**Supplementary material**

|  | **Observer 1** | **Observer 2** | **Intra observer**  **Relative bias (%) /**  **bias SD /**  **coefficient of variation** | **Inter observer**  **Relative bias (%) /**  **bias SD /**  **coefficient of variation** |
| --- | --- | --- | --- | --- |
| T1 ROI 1 (ms) | 734± 84 | 726±62 | 0.6/ 28.2/ 3.9 | 2.3/ 53.0/ 7.2 |
| T1 ROI 2 (ms) | 677± 66 | 684±62 | 0.5/ 33.2/ 4.9 | -1.0/ 30.1/ 4.4 |
| T1 ROI 3 (ms) | 722± 71 | 726±67 | -1.0/ 30.2/ 4.2 | -0.6/ 37.3/ 5.1 |
| T1 average  ROI 1-3 (ms) | 711± 66 | 709±59 | 0.1/ 16.1/ 2.3 | 0.2/ 19.6/ 2.8 |
| ECV ROI 1 (%) | 39.5± 6.4 | 38.1±5.7 | 3.2/ 6.2/ 16.0 | 3.4/ 5.0/ 12.8 |
| ECV ROI 2 (%) | 34.7± 4.8 | 35.9±5.1 | 5.3/ 4.4/ 12.9 | -3.3/ 5.2/ 14.9 |
| ECV ROI 3 (%) | 39.4± 4.6 | 40.0±5.6 | 1.2/ 3.8/ 9.8 | -1.4/ 4.6/ 11.5 |
| ECV average  ROI 1-3 (%) | 37.0± 4.4 | 38.7.0±4.7 | 3.2/ 4.0/ 10.7 | -2.3/ 4.2/ 10.9 |

Supplementary Table 1. Inter region T1, ECV differences.

Native T1 times and extracellular volume fraction (ECV) for the different ROIs 1-3 and the average of ROIs 1-3 with observer variation.
